# Supplementary material for: Modeling glioblastoma heterogeneity as a dynamic network of cell states
Source: Mol Syst Biol. 2021 Sep 16;17(9):e10105. doi: 10.15252/msb.202010105 (PMC8444284; doi:10.15252/msb.202010105)
Supplement: Supplementary file 5 — Source Data for Figure 3 [file MSB-17-e10105-s001.zip › Figure3A_sourcedata/GSEA_3065/hallmarks_state2.GseaPreranked.1623416269049/HALLMARK_ADIPOGENESIS.html]

Details for gene set HALLMARK\_ADIPOGENESIS[GSEA]

|  || Dataset | state2 |
| Phenotype | NoPhenotypeAvailable |
| Upregulated in class | na\_pos |
| GeneSet | HALLMARK\_ADIPOGENESIS |
| Enrichment Score (ES) | 0.21764098 |
| Normalized Enrichment Score (NES) | 0.7642374 |
| Nominal p-value | 0.92108506 |
| FDR q-value | 0.88481754 |
| FWER p-Value | 1.0 |
Table: GSEA Results Summary

  

Fig 1: Enrichment plot: HALLMARK\_ADIPOGENESIS      
 Profile of the Running ES Score & Positions of GeneSet Members on the Rank Ordered List

  

| PROBE | GENE SYMBOL | GENE\_TITLE | RANK IN GENE LIST | RANK METRIC SCORE | RUNNING ES | CORE ENRICHMENT || 1 | CDKN2C |  |  | 123 | 0.341 | 0.0167 | Yes |
| 2 | COX8A |  |  | 414 | 0.197 | 0.0028 | Yes |
| 3 | GPX4 |  |  | 425 | 0.194 | 0.0187 | Yes |
| 4 | UQCRQ |  |  | 501 | 0.181 | 0.0265 | Yes |
| 5 | CYC1 |  |  | 520 | 0.178 | 0.0402 | Yes |
| 6 | MTCH2 |  |  | 557 | 0.171 | 0.0513 | Yes |
| 7 | MDH2 |  |  | 615 | 0.161 | 0.0593 | Yes |
| 8 | ACADM |  |  | 715 | 0.148 | 0.0616 | Yes |
| 9 | HADH |  |  | 778 | 0.141 | 0.0674 | Yes |
| 10 | ATP5PO |  |  | 804 | 0.138 | 0.0768 | Yes |
| 11 | PRDX3 |  |  | 877 | 0.130 | 0.0804 | Yes |
| 12 | UQCR10 |  |  | 941 | 0.125 | 0.0846 | Yes |
| 13 | AK2 |  |  | 948 | 0.124 | 0.0948 | Yes |
| 14 | TALDO1 |  |  | 954 | 0.123 | 0.1051 | Yes |
| 15 | NDUFAB1 |  |  | 970 | 0.122 | 0.1142 | Yes |
| 16 | IMMT |  |  | 988 | 0.121 | 0.1230 | Yes |
| 17 | SOD1 |  |  | 1028 | 0.117 | 0.1291 | Yes |
| 18 | G3BP2 |  |  | 1030 | 0.117 | 0.1393 | Yes |
| 19 | COX7B |  |  | 1053 | 0.115 | 0.1470 | Yes |
| 20 | SUCLG1 |  |  | 1076 | 0.113 | 0.1545 | Yes |
| 21 | MGST3 |  |  | 1210 | 0.104 | 0.1493 | Yes |
| 22 | NDUFS3 |  |  | 1288 | 0.099 | 0.1497 | Yes |
| 23 | ECHS1 |  |  | 1322 | 0.097 | 0.1546 | Yes |
| 24 | UCP2 |  |  | 1329 | 0.096 | 0.1624 | Yes |
| 25 | ETFB |  |  | 1334 | 0.096 | 0.1704 | Yes |
| 26 | SDHB |  |  | 1429 | 0.091 | 0.1682 | Yes |
| 27 | VEGFB |  |  | 1490 | 0.087 | 0.1694 | Yes |
| 28 | DDT |  |  | 1544 | 0.084 | 0.1711 | Yes |
| 29 | NDUFB7 |  |  | 1596 | 0.082 | 0.1728 | Yes |
| 30 | CMPK1 |  |  | 1647 | 0.079 | 0.1744 | Yes |
| 31 | GHITM |  |  | 1661 | 0.079 | 0.1799 | Yes |
| 32 | GRPEL1 |  |  | 1664 | 0.079 | 0.1866 | Yes |
| 33 | MRPL15 |  |  | 1714 | 0.076 | 0.1880 | Yes |
| 34 | GADD45A |  |  | 1791 | 0.073 | 0.1862 | Yes |
| 35 | UQCR11 |  |  | 1831 | 0.071 | 0.1882 | Yes |
| 36 | SLC25A10 |  |  | 1847 | 0.070 | 0.1928 | Yes |
| 37 | ACAA2 |  |  | 1924 | 0.067 | 0.1905 | Yes |
| 38 | UCK1 |  |  | 1968 | 0.066 | 0.1917 | Yes |
| 39 | PIM3 |  |  | 1972 | 0.066 | 0.1971 | Yes |
| 40 | PPP1R15B |  |  | 2009 | 0.064 | 0.1989 | Yes |
| 41 | AIFM1 |  |  | 2050 | 0.063 | 0.2001 | Yes |
| 42 | RAB34 |  |  | 2057 | 0.062 | 0.2049 | Yes |
| 43 | GPD2 |  |  | 2101 | 0.061 | 0.2056 | Yes |
| 44 | IDH3A |  |  | 2185 | 0.058 | 0.2018 | Yes |
| 45 | COQ9 |  |  | 2199 | 0.057 | 0.2054 | Yes |
| 46 | SLC25A1 |  |  | 2256 | 0.055 | 0.2042 | Yes |
| 47 | CS |  |  | 2262 | 0.055 | 0.2085 | Yes |
| 48 | MGLL |  |  | 2293 | 0.054 | 0.2100 | Yes |
| 49 | YWHAG |  |  | 2297 | 0.054 | 0.2144 | Yes |
| 50 | QDPR |  |  | 2314 | 0.053 | 0.2174 | Yes |
| 51 | UQCRC1 |  |  | 2355 | 0.052 | 0.2176 | Yes |
| 52 | SAMM50 |  |  | 2471 | 0.049 | 0.2095 | No |
| 53 | PFKL |  |  | 2489 | 0.048 | 0.2119 | No |
| 54 | ARL4A |  |  | 2510 | 0.047 | 0.2139 | No |
| 55 | NDUFA5 |  |  | 2662 | 0.043 | 0.2014 | No |
| 56 | SOWAHC |  |  | 2668 | 0.043 | 0.2046 | No |
| 57 | DECR1 |  |  | 2696 | 0.042 | 0.2053 | No |
| 58 | CRAT |  |  | 2856 | 0.038 | 0.1915 | No |
| 59 | DLD |  |  | 2865 | 0.037 | 0.1939 | No |
| 60 | UBQLN1 |  |  | 2923 | 0.036 | 0.1909 | No |
| 61 | ALDH2 |  |  | 2958 | 0.035 | 0.1903 | No |
| 62 | COQ3 |  |  | 2988 | 0.034 | 0.1902 | No |
| 63 | DLAT |  |  | 3116 | 0.031 | 0.1793 | No |
| 64 | DNAJC15 |  |  | 3482 | 0.024 | 0.1421 | No |
| 65 | ADIPOR2 |  |  | 3549 | 0.023 | 0.1370 | No |
| 66 | CHUK |  |  | 3569 | 0.022 | 0.1369 | No |
| 67 | PEMT |  |  | 3847 | 0.018 | 0.1087 | No |
| 68 | COQ5 |  |  | 3856 | 0.018 | 0.1094 | No |
| 69 | BCL2L13 |  |  | 3857 | 0.018 | 0.1110 | No |
| 70 | PGM1 |  |  | 3936 | 0.016 | 0.1040 | No |
| 71 | ECH1 |  |  | 4011 | 0.015 | 0.0974 | No |
| 72 | LAMA4 |  |  | 4068 | 0.014 | 0.0926 | No |
| 73 | NMT1 |  |  | 4164 | 0.013 | 0.0835 | No |
| 74 | PREB |  |  | 4188 | 0.012 | 0.0821 | No |
| 75 | IDH3G |  |  | 4210 | 0.012 | 0.0809 | No |
| 76 | RNF11 |  |  | 4387 | 0.009 | 0.0627 | No |
| 77 | ALDOA |  |  | 4435 | 0.008 | 0.0584 | No |
| 78 | PHYH |  |  | 4479 | 0.008 | 0.0544 | No |
| 79 | JAGN1 |  |  | 4684 | 0.004 | 0.0329 | No |
| 80 | TST |  |  | 4725 | 0.004 | 0.0289 | No |
| 81 | PLIN2 |  |  | 4837 | 0.002 | 0.0171 | No |
| 82 | SLC5A6 |  |  | 5054 | -0.001 | -0.0061 | No |
| 83 | GPAT4 |  |  | 5183 | -0.003 | -0.0196 | No |
| 84 | MCCC1 |  |  | 5214 | -0.004 | -0.0225 | No |
| 85 | TANK |  |  | 5234 | -0.004 | -0.0242 | No |
| 86 | SDHC |  |  | 5266 | -0.004 | -0.0272 | No |
| 87 | DHRS7B |  |  | 5267 | -0.004 | -0.0268 | No |
| 88 | NKIRAS1 |  |  | 5268 | -0.004 | -0.0264 | No |
| 89 | SLC19A1 |  |  | 5319 | -0.005 | -0.0314 | No |
| 90 | PPM1B |  |  | 5399 | -0.007 | -0.0393 | No |
| 91 | CMBL |  |  | 5463 | -0.008 | -0.0454 | No |
| 92 | SLC1A5 |  |  | 5584 | -0.010 | -0.0575 | No |
| 93 | LPCAT3 |  |  | 5624 | -0.010 | -0.0608 | No |
| 94 | GPAM |  |  | 5678 | -0.011 | -0.0655 | No |
| 95 | ARAF |  |  | 5785 | -0.013 | -0.0758 | No |
| 96 | FAH |  |  | 5837 | -0.014 | -0.0800 | No |
| 97 | SCP2 |  |  | 5857 | -0.014 | -0.0809 | No |
| 98 | SSPN |  |  | 5862 | -0.014 | -0.0801 | No |
| 99 | CPT2 |  |  | 5964 | -0.016 | -0.0895 | No |
| 100 | ACO2 |  |  | 6010 | -0.017 | -0.0929 | No |
| 101 | PTCD3 |  |  | 6180 | -0.020 | -0.1094 | No |
| 102 | IDH1 |  |  | 6208 | -0.020 | -0.1105 | No |
| 103 | ESRRA |  |  | 6213 | -0.020 | -0.1092 | No |
| 104 | DBT |  |  | 6309 | -0.022 | -0.1175 | No |
| 105 | DRAM2 |  |  | 6501 | -0.025 | -0.1359 | No |
| 106 | RMDN3 |  |  | 6704 | -0.030 | -0.1550 | No |
| 107 | SCARB1 |  |  | 6752 | -0.031 | -0.1574 | No |
| 108 | AGPAT3 |  |  | 6767 | -0.031 | -0.1562 | No |
| 109 | GBE1 |  |  | 6821 | -0.032 | -0.1591 | No |
| 110 | CHCHD10 |  |  | 6924 | -0.035 | -0.1671 | No |
| 111 | MAP4K3 |  |  | 7016 | -0.037 | -0.1736 | No |
| 112 | CAT |  |  | 7094 | -0.039 | -0.1785 | No |
| 113 | DGAT1 |  |  | 7121 | -0.040 | -0.1778 | No |
| 114 | RIOK3 |  |  | 7185 | -0.041 | -0.1810 | No |
| 115 | ELMOD3 |  |  | 7282 | -0.044 | -0.1874 | No |
| 116 | ACLY |  |  | 7320 | -0.045 | -0.1874 | No |
| 117 | CCNG2 |  |  | 7353 | -0.046 | -0.1868 | No |
| 118 | MIGA2 |  |  | 7375 | -0.047 | -0.1850 | No |
| 119 | BAZ2A |  |  | 7378 | -0.047 | -0.1811 | No |
| 120 | COX6A1 |  |  | 7383 | -0.047 | -0.1774 | No |
| 121 | PDCD4 |  |  | 7568 | -0.052 | -0.1926 | No |
| 122 | STOM |  |  | 7570 | -0.052 | -0.1881 | No |
| 123 | BCL6 |  |  | 7753 | -0.058 | -0.2026 | No |
| 124 | DNAJB9 |  |  | 7882 | -0.062 | -0.2109 | No |
| 125 | ANGPT1 |  |  | 7891 | -0.063 | -0.2063 | No |
| 126 | IFNGR1 |  |  | 7945 | -0.065 | -0.2063 | No |
| 127 | HIBCH |  |  | 8030 | -0.068 | -0.2094 | No |
| 128 | LPL |  |  | 8087 | -0.071 | -0.2092 | No |
| 129 | ABCB8 |  |  | 8099 | -0.071 | -0.2041 | No |
| 130 | LIFR |  |  | 8134 | -0.073 | -0.2013 | No |
| 131 | TOB1 |  |  | 8169 | -0.076 | -0.1984 | No |
| 132 | ADCY6 |  |  | 8196 | -0.077 | -0.1944 | No |
| 133 | ESYT1 |  |  | 8283 | -0.082 | -0.1965 | No |
| 134 | TKT |  |  | 8389 | -0.088 | -0.2001 | No |
| 135 | SLC27A1 |  |  | 8407 | -0.089 | -0.1940 | No |
| 136 | ITSN1 |  |  | 8414 | -0.090 | -0.1868 | No |
| 137 | ACOX1 |  |  | 8525 | -0.098 | -0.1900 | No |
| 138 | SORBS1 |  |  | 8562 | -0.101 | -0.1851 | No |
| 139 | ATL2 |  |  | 8565 | -0.101 | -0.1764 | No |
| 140 | PHLDB1 |  |  | 8610 | -0.104 | -0.1720 | No |
| 141 | ABCA1 |  |  | 8635 | -0.106 | -0.1653 | No |
| 142 | DHRS7 |  |  | 8745 | -0.116 | -0.1669 | No |
| 143 | MYLK |  |  | 8862 | -0.131 | -0.1678 | No |
| 144 | RTN3 |  |  | 8926 | -0.138 | -0.1625 | No |
| 145 | UBC |  |  | 8968 | -0.144 | -0.1543 | No |
| 146 | DHCR7 |  |  | 9009 | -0.150 | -0.1454 | No |
| 147 | ITGA7 |  |  | 9011 | -0.150 | -0.1324 | No |
| 148 | POR |  |  | 9033 | -0.155 | -0.1210 | No |
| 149 | ELOVL6 |  |  | 9070 | -0.161 | -0.1108 | No |
| 150 | CAVIN1 |  |  | 9071 | -0.161 | -0.0966 | No |
| 151 | CD151 |  |  | 9094 | -0.165 | -0.0845 | No |
| 152 | REEP5 |  |  | 9177 | -0.184 | -0.0772 | No |
| 153 | COL4A1 |  |  | 9284 | -0.226 | -0.0688 | No |
| 154 | ATP1B3 |  |  | 9352 | -0.274 | -0.0519 | No |
| 155 | SPARCL1 |  |  | 9360 | -0.288 | -0.0274 | No |
| 156 | APLP2 |  |  | 9409 | -0.408 | 0.0033 | No |
Table: GSEA details [plain text format]

  

Fig 2: HALLMARK\_ADIPOGENESIS: Random ES distribution      
 Gene set null distribution of ES for **HALLMARK\_ADIPOGENESIS**
